# COVID-19 Social Restrictions: An Opportunity to Re-visit the Concept of Harm Reduction in the Treatment of Alcohol Dependence. A Position Paper

**DOI:** 10.3389/fpsyt.2021.623649

**Published:** 2021-02-18

**Authors:** Christos Kouimtsidis, Bernadette Pauly, Tessa Parkes, Tim Stockwell, Alexander Mario Baldacchino

**Affiliations:** ^1^Imperial College London and Surrey and Borders Partnership NHS Foundation Trust, London, United Kingdom; ^2^Canadian Institute for Substance Use Research, University of Victoria School of Nursing, Victoria, BC, Canada; ^3^Faculty of Social Sciences, University of Stirling, Stirling, United Kingdom; ^4^University of Victoria, Canadian Institute for Substance Use Research, Victoria, BC, Canada; ^5^Population and Behavioural Science Division, Medical School, St Andrews University, St Andrews, United Kingdom

**Keywords:** harm reduction, structured preparation for alcohol detoxification, managed alcohol programs, alcohol, COVID-19

## Abstract

The COVID-19 pandemic is presenting significant challenges for health and social care systems globally. The implementation of unprecedented public health measures, alongside the augmentation of the treatment capacity for those severely affected by COVID-19, are compromising and limiting the delivery of essential care to people with severe substance use problems and, in some cases, widening extreme social inequities such as poverty and homelessness. This global pandemic is severely challenging current working practices. However, these challenges can provide a unique opportunity for a flexible and innovative learning approach, bringing certain interventions into the spotlight. Harm reduction responses are well-established evidenced approaches in the management of opioid dependence but not so well-known or implemented in relation to alcohol use disorders. In this position paper, we explore the potential for expanding harm reduction approaches during the COVID-19 crisis and beyond as part of substance use treatment services. We will examine alcohol use and related vulnerabilities during COVID-19, the impact of COVID-19 on substance use services, and the potential philosophical shift in orientation to harm reduction and outline a range of alcohol harm reduction approaches. We discuss relevant aspects of the Structured Preparation for Alcohol Detoxification (SPADe) treatment model, and Managed Alcohol Programs (MAPs), as part of a continuum of harm reduction and abstinence orientated treatment for alcohol use disorders. In conclusion, while COVID-19 has dramatically reduced and limited services, the pandemic has propelled the importance of alcohol harm reduction and created new opportunities for implementation of harm reduction philosophy and approaches, including programs that incorporate the provision of alcohol as medicine as part of the substance use treatment continuum.

## Introduction

On March 11, 2020 the World Health Organization declared a global pandemic due to the novel coronavirus (1). The call rippled globally resulting in the implementation of public health measures including travel restrictions, stay-at-home orders, frequent handwashing, physical distancing, and self-isolation (2). COVID-19 has dramatic implications for those with alcohol use disorders (AUD) due to changes in the severity and pattern of drinking, changes in access to services with restrictions and closures, as well as significant shifts in the mode of delivery of substance use services (3, 4). The pandemic demands attention to the continuum of substance use services, including alcohol harm reduction, and the specific needs of those impacted by intersecting crises of alcohol use disorders, poverty and homelessness (5, 6).

Harm reduction to prevent the transmission of blood borne diseases, prevent overdoses, and provide an alternative to an unsafe illicit drug supply, is underpinned by the goal of reducing harm associated with illicit drug use (7, 8). Alcohol harm reduction, like other harm reduction approaches, aims to reduce the harms of alcohol without necessarily requiring a reduction in, or stopping, drinking (9). Strategies to reduce harm from alcohol often focus on general population strategies, such as low risk drinking guidelines and population-based policies related to pricing and other forms of regulation, to reduce overall population harm. While critically important to population health, this approach is not sufficient to reduce individual harms for some groups, and may even have unintended consequences that increase harms (8). While there is robust evidence for interventions to reduce harms of illicit drug use, much less attention has been paid to reducing the many harms associated with alcohol use, specifically heavy episodic drinking, chronic use, and illicit and non-beverage alcohol use. Alcohol harm reduction for individuals this includes pharmaceutical alternatives to reduce cravings, potential use of cannabis as a substitution for alcohol, social interventions such as Housing First programs where substance use including alcohol use is tolerated, safer drinking education, and programs that provide alcohol. During the COVID-19 pandemic, the importance of alcohol harm reduction as an adjunct to other approaches has become increasingly prominent due to changes in service provision.

In this position paper, our objectives are to: (i) explore the shifts in relation to harms associated with AUD during COVID-19; (ii) illustrate both adverse and optimal changes in substance use and addiction services during the pandemic, and; (iii) underscore the philosophical shifts and opportunities for enhancing harm reduction strategies for those with AUD during the pandemic and beyond. We draw on international literature, wherever available, with specific examples from the UK and Canada. We did not undertake a systematic search of the literature but team members collated specific COVID-19 and AUD publications throughout the pandemic, most specifically utilizing the Society for the Study of Addiction COVID-19 research/briefings/evidence web-based resource (10). Our aim is to highlight harm reduction as an important approach and set of strategies for reducing alcohol related harms as part of health care systems and alongside treatment services during COVID 19 and beyond.

## Alcohol Related Harms and Vulnerability

In 2016, the use of alcohol was estimated to result in 2.8 million deaths (5.3% of all deaths) worldwide and 132.6 million disability-adjusted life years (DALYs) (11). Alcohol related mortality exceeds that caused by other communicable and non-communicable diseases, such as tuberculosis, HIV/AIDS and diabetes. Harms from alcohol and other drugs can be classified into those which are: (i) “acute,” comprising injuries, poisonings and/or acute illnesses partly caused by an episode of heavy use; (ii) “chronic,” comprising a range of chronic and relapsing conditions including liver disease, cancers, strokes and gastrointestinal diseases which are caused by the overall volume of alcohol consumed over time (12), and; (iii) “social,” which may involve problems in the spheres of housing, finances, relationships, the law, and workplace (12). Contextually, harms are increased along the socio-economic gradient, with increased alcohol-related harms experienced by those with low socio-economic status (13–16).

AUDs are experienced by 3–4% of the population globally (17). DSM-V includes dependence under the category of AUD and is defined as a clustering of signs of increased tolerance, the experience of withdrawal, continued use despite the experience of problems, and a degree of impaired control over consumption (18). Alcohol dependence carries heavy health and social costs which are increased when associated with poverty, homelessness, and/or housing instability (19–21). An international review found 10 studies concerning severe AUD experienced by men who are homeless but little data was available for homeless women (22). Among homeless men in economically developed countries, the prevalence of severe AUDs has been estimated to be almost 40% (22). Homelessness is associated with higher rates of depression, suicide, chronic pain, and poor mental health, alongside inadequate housing, food and other insecurities, as a consequence of severe poverty (23–26). The combination of severe AUDs and homelessness is often a response to, and a consequence of, multiple intersecting structural, systemic, and individual factors, in which alcohol can be a means of coping (27–29).

The relationship between stress and alcohol use is bilateral. Stress has been recognized as a predisposing risk factor for the development of AUD, and chronic alcohol use can exaggerate the experience of stress and compromise the ability of the individual to cope with stress (30). In the early stages of the pandemic, two opposite scenarios were introduced based on a review of the impact of previous epidemics by Rehm et al. (31). The first scenario predicted an increase of consumption, in particular in men, due to increased stress, and the second scenario predicted a reduction of consumption due to a reduction of access to alcohol, due to the social distancing measures (31). In fact, policymakers in many countries deemed alcohol sale to be “essential” and loosened alcohol restrictions e.g., allowing internet orders and home delivery. It seems that two main factors [vulnerability to stress and increased access to alcohol), had a synergic impact that further exaggerated pre-exisiting vulnerabilities of people with AUD (4). There is emerging evidence that alcohol use increased during the early phase of the pandemic, in both the general population (32, 33) and the population with pre-exisiting AUD (34)]. It has been also documented that limited access to alcohol led to increased frequency of abrupt discontinuation, and a temporary uptick in presentations to hospital for management of alcohol withdrawal symptoms experienced by dependent and heavy drinkers (4). This change in consumption, in conjunction with limited access to generic health and specialist services, plus the impact of the pandemic on social care and social stability, suggest the need to review the potential usefulness of alcohol harm reduction strategies during the COVID-19 period.

## Changes to Service Provision During COVID 19 for People With AUD

COVID-19 has affected every healthcare system in the world, even in countries that have not had high numbers of COVID-19 case numbers. According to Sutherland et al. [(35), p8], “*different healthcare systems have seen varying patterns of changes in healthcare activity – depending on prevalence, the stage of the pandemic and local policy.”* Preparations to help health and social care services cope with anticipated increased demand from patients with severe cases of COVID-19, and the requirement to reduce the risk of infection/transmission, led to tremendous global changes in health service provision for non-COVID-19 related conditions, and also to public expectations of what would be provided by healthcare services (4). For example, Sutherland et al. (35) investigated changes in New South Wales, Australia using healthcare data drawn from multiple sources. Their study found that, between March and June 2020, compared with the same period in 2019, primary care face-to-face consultations decreased by 22.1%, breast screening activity by 51.5%, ambulance incidents by 7.2%, emergency department visits by 13.9%, public hospital inpatient episodes by 14.3%, and public hospital planned surgical activity by 32.6%. They concluded that there were substantial declines in a wide range of healthcare activities across the NSW health system over this period and, while activity was recovering by September 2020, they had still not returned to “normal.” There was widespread deferment of scheduled appointments and procedures to attempt to accommodate the actual or predicted COVID-19 cases.

Across the world, staff were redeployed to unfamiliar environments away from services deemed non-essential to the COVID-19 response (36). This also involved the need for retraining and repurposing of staff resources. In England, for example, new staff such as trainees in the early stages of their career (foundation and core trainees), retired colleagues, or staff from other hospital departments, were deployed to increase capacity within emergency departments. These staff might not have been aware of existing protocols for cross departmental coordination, coordination with primary care, or secondary care specialist services such as drug and alcohol services. Restrictions of provision of substance use hospital liaison services was also experienced. This was due in part to generic measures employed to protect staff (rotation of work force or over the phone advice), as well as re-deployment of acute hospital staff, such as phlebotomists, clinical and administrative staff. This led to major reductions in/lack of access to services such as provision of liver function tests and regular hepatology outpatient appointments (37). Another important factor during the initial period of the COVID-19 response was fear on behalf of the public regarding the risk of infection if they approached health services impacting on seeking help for non-COVID-19 conditions, and a reluctance to place additional burden on health care services (35). For people with AUD, this could exacerbate pre-existing fragmentation in service provision and contribute to the long term deterioration of health and unnecessary therapeutic pessimism (4). Services were also reconfigured to accommodate the need for physical distancing, for example by moving services on to virtual platforms (35). The above mentioned barriers are increased for people impacted by severe AUD, poverty and/or homelessness, who may lack access to primary care.

### Changes to Specialist Substance Use/Addiction Services

As documented during a temporary alcohol prohibition in India (38), temporary spikes in treatment seeking for alcohol withdrawal may occur initially but these rapidly decline, as has also been documented during other major alcohol restrictions (39). There is a complex interplay over time between alcohol supply and alcohol harm. During COVID 19, requirements for social distancing introduced by most countries have led to major changes to substance use specialist service provision. The most common changes adopted across a range of countries were (i) stopping provision of treatment *via* structured group work, (ii) stopping community detoxification, and (iii) reduction of face to face consultation to the minimum and, in some cases, reduced access to withdrawal management and rehabilitation services (36, 40). These changes have disproportionally affected substance use service provision for individuals with AUDs (39, 41). In some countries, addiction/substance use services were deemed to be essential services and thus protected from having staff resource redeployed (36). It is important to note that, while there were extensive clinical guidelines and advice being issued early in the pandemic to provide continuity of service and contingency planning (36), it was hard for service providers to adapt quickly while also continuing to provide services (6, 42–44). There is also the risk that the most vulnerable sub group of people with AUD, such as those experiencing homelessness and unemployment, would not necessarily have the technology to be able to access virtual services offered by phone or computer (5, 6). There are examples of attempts to address those barriers, for example in Scotland where phones were distributed to this group to address digital barriers (45). This population also lost other community supports such as access to food banks, due to reduced capacity and requirements for social distancing (6) and for some loss of income from begging/panning and recycling.

### Mental Health Impact and Access to Mental Health Services

The mental health impact of various elements of the pandemic on the general population and on people with pre-exisiting mental health conditions was acknowledged early on by the scientific community (46–50). Similar impacts were therefore expected for vulnerable people with substance use problems such as AUD (36). According to DeJong et al. (36) who conducted a qualitative study with people in substance use treatment including for alcohol problems in the Netherlands, COVID-19 feelings of anger, guilt, gloom, fear, panic, restlessness, and stress were reported by participants, along with social isolation, lack of structure and boredom. The additional stress of a pandemic can create additional vulnerabilities in relation to physical and psychological health (51), and also increase risk of relapse (52). Increased levels of stress due to fear of infection, illness and death, as well as financial stressors, can increase levels of stress experienced by an already vulnerable population with AUD that is additionally compromised due to chronicity of drinking (36).

### Social Care and Community Services

Prior to COVID-19, individuals with both severe AUD and homelessness faced significant barriers to accessing temporary accommodation and, in some cases, had to go without shelter as a consequence of alcohol use (53). Pre-existing structural vulnerability and alcohol related harms for this population were escalated with the announcement of the global pandemic in March 2020. Individuals may also have had difficulties accessing beverage alcohol due to restricted hours, restrictions on the use of cash, and implementation of isolation measures and restrictive policies that limited guests or public access (6). Additionally, socioeconomic factors may affect purchasing ability, such as loss of income from begging, pan handling, and closure of bottle or recycling depots (41). These factors may shift patterns of drinking in ways that increase harms, or lead to other unanticipated consequences, such as alcohol withdrawal, alcohol poisoning and/or substitution of illicit drugs for alcohol. Due to costs and availability, use of non-beverage alcohol such as hand sanitizer and rubbing alcohol can increase among those who are homeless posing significant harms (54). Also, this group may experience more serious COVID-19 symptoms due to the higher risk of pneumonia and compromised immune function associated with high levels of alcohol consumption (55). Further, the requirements of physical distancing and self-isolation may contribute to even greater social isolation, marginalization, and loss of social networks.

## Strategic Change in Treatment Philosophy Toward Harm Reduction

A harm reduction approach, beyond the provision of safety from unwanted withdrawals, that can be combined with other treatment components across a range of settings, such as emergency departments, primary care and specialist community services, became necessary during the pandemic. Phone and digital consultations were widely used during this period to support clients in opioid substitution treatment, alongside other measures and modifications compatible with social distancing. For individuals with AUDs, however, where substitution was not an option, digital or phone consultations, might not be sufficient, whilst other components of the treatment pathway, such as detoxification and group work, are interrupted. To maximize their effectiveness these consultations should be planned and structured with the aim of maintaining a therapeutic component (56).

A harm reduction approach, informed by the changes required during the COVID-19 pandemic, as applied in harm reduction for opioid treatment (57), is therefore needed. Managing risks around COVID-19 could mean self-isolation and reduction of income which, in turn, might put the ability of the person to maintain stable levels and patterns of drinking at risk. This may increase risk of severe withdrawal complications (e.g., seizures) (4, 58). Harm reduction advice to maintain stable levels of drinking, while facilitating engagement with AUD services, could be expanded in conjunction with AUD services, given the lack of access to community detoxification, acute hospital admission, or reduced access to inpatient specialist detoxification services. However, expansion of alcohol supply has to always be carefully balanced against the high level of demonstrable harm to health attributed to alcohol, with rates of associated morbidity, mortality and economic costs far higher than for other substances (59).

The harm reduction approach is compatible with an overall pre-habilitation approach to the management of AUD. Pre-habilitation advocates the identification and proactive management of; (i) any factors anticipated to compromise the successful outcome of an intervention, and; (ii) the potential side effects associated with the intervention itself. It is a pro-active rather than a reactive approach aimed at ensuring more sustainable outcomes (60). Harm reduction, using alcohol as an agent of treatment, could achieve both aims (54). The concept of pre-habilitation is not new. The ability to predict, or anticipate, certain harm, or assess certain risks, is associated with the human ability of learning from experience, modifying behavioral responses, and developing long-term and sustainable response strategies. To that effect, planning in advance, in anticipation of risks, can be considered to be an essential strategy and quality, associated with individual survival and progress.

## Potential Harm Reduction Strategies Within a Harm Reduction Framework for People With AUD

Alcohol harm reduction for individual clients refers to a range of strategies and approaches that specifically seek to reduce the harms of alcohol without necessarily requiring a reduction in, or stopping, drinking. Specific alcohol harm reduction strategies include: (1) use of pharmaceutical alternatives to reduce cravings; (2) use of cannabis as a substitution for alcohol; (3) social interventions such as Housing First programs where substance use and alcohol use is tolerated; (4) safer drinking education; (5) substitution programs that provide alcohol. Although individuals experiencing severe AUD and homelessness often express a preference for harm reduction goals, there is limited discussion and availability of specific alcohol harm reduction strategies (61–63). We will provide a brief overview of the first four strategies and provide more detail on substitution programs that provide alcohol, such as SPADE and MAP, as forms of alcohol harm reduction that could be enhanced in substance use services. We will comment on the need for the strategy during COVID 19 and any particular challenges and adaptations that the COVID-19 pandemic might necessitate to those strategies.

### Pharmaceutical Alternatives

Pharmaceutical alternatives include use of medication such as Naltrexone or Acamposate to manage craving and withdrawal symptoms, and may be used alone or in combination with other approaches such as motivational interviewing. Different medications are approved for use in different countries (58). Limited access to health services due to the pandemic (as discussed in section Social Care and Community Services above) might have reduced capacity for baseline and ongoing monitoring such as liver function tests, necessitating adaptation of the clinical protocols to pandemic mode.

### Cannabis Substitution

Cannabis use has also been suggested as a substitute for alcohol. Where abstinence is neither feasible or preferable, cannabis has been used within a harm reduction framework to reduce use of other substances and help meet goals of reducing harm (64–66). In particular, cannabis substitution has been proposed as a potential harm reduction strategy for those with alcohol dependence (67). Cannabis substitution for alcohol problems meets, or partially meets, the seven criteria for evaluating the use of substitution medicines developed by Chick and Nutt (68). The need for further evidence through clinical trials has been recommended (68). While cannabis use is not without harm, it is argued that the scale of harms is substantially lower than for alcohol (68, 69). It has the potential to stave off cravings and reduce withdrawal, as well as having a potential beneficial effect for pain, PTSD, anxiety, and sleep (69). However, cannabis can potentiate the effects of alcohol and more evidence is needed as to its use and effectiveness with people with AUD. During COVID-19, especially in the context of legalization of cannabis, or in a medical context, such a strategy could be considered harm reduction where other interventions are not accessible or unacceptable, and with appropriate guidelines for safe use.

### Housing First

Tolerance of substance use in Housing First programs has been associated with improved costs and better outcomes for those able to manage their own alcohol use (70–73). This strategy is even more crucial during the period of the COVID-19 pandemic as housing is a front line defense against COVID-19. There are indications that the financial and social impact associated with the measures taken to manage the pandemic has increased unemployment, loss of income, and in some cases homelessness (74).

### Safer Drinking Education

In some cases, safer drinking education has been incorporated as an intervention in Housing First programs to reduce harms. Safer drinking education includes provision of information and education by peers that focuses on reduces the risks of drinking (75). This approach could be used in a wide range of community settings including outreach, shelters and drop-ins. Specifically, two of the authors led the development of safer drinking tips during COVID 19.

### Substitution Programs That Provide Beverage Alcohol

The principles of a harm reduction approach that helps people who use opioids to stay alive and, safe, and which provides easy access into other components of treatment, has relevance for people with AUD. While there is no substitution substance for alcohol, managed access to beverage alcohol has been provided by Managed Alcohol Programs (MAP)s in Canada, often to replace use of non-beverage alcohol, which may both be more intrinsically harmful, and easier to consume in harmful quantities due to higher alcohol concentrations and lower prices. Structured Preparation for Alcohol Detoxification (SPADe) and MAP are now examined in more depth as harm reduction approaches that provide alcohol as medication within a harm reduction framework which, during the COVID-19 period, can reduce the risk and severity of abrupt and unplanned withdrawal, as well as harms related to use of non-beverage alcohol.

### Structured Preparation for Alcohol Detoxification (SPADe)

The emphasis of SPADe is on stable drinking and avoidance of major fluctuations in the amount and pattern of drinking as the first step toward preparation for abstinence, as well as a final aim for controlled drinking. The SPADe approach, although not described as “harm reduction” *per se*, has similar components to a harm reduction approach, given that it promotes the use of alcohol as a medication, with frequent and regular dosing to prevent rather than treat withdrawal symptoms. Within SPADe, the main aim is the stabilization of both the amount and pattern of drinking. This type of controlled drinking is referred to as “partial” for two main reasons: (a) it is an intermediate treatment stage rather than the final treatment aim, which remains abstinence and; (b) the amount and pattern of drinking during this process is not always within healthy limits (76).

This proactive elimination of symptoms is considered fundamental from a biological perspective as it protects against brain acute dysregulation which, in turn, might sensitize the brain, leading to an exaggeration of the negative impact associated with the disturbance of the brain's homeostatic system. From a psychological perspective, it empowers the individual through regaining some control of decision making, thus reducing the impulsivity associated with the experience or avoidance of experiencing cravings and withdrawal symptoms. Furthermore, it provides a relatively stable environment for the individual and the close social environment to start implementing lifestyle changes leading to increased self-efficacy which is considered to be the final mediating factor in social learning theory and cognitive and behavioral treatment models (77, 78).

The amount of drinking, following stabilization of the patterns described above, could be reduced gradually following the principle of small sustainable changes. The aim is to avoid any dramatic change to the amount of drinking that might not only be unsustainable but also lead to precipitation of withdrawal symptoms which, on rare occasions, might potentially be life threatening. Once stability is achieved then gradual reduction can be safely initiated. Roughly half of the individuals would be able to stop using alcohol without the use of detoxification medication (79). This model of detoxification is called “guided self-detox” and refers to the process of using alcohol “as if it was medication” and as a safe detoxification tool. During the period of COVID-19 pandemic, with the associated limitations in specialist service provision, the stabilization of drinking and the guided self-detox wherever possible, rather than detoxification seems to be a safer and more realistic treatment aim.

Within the SPADe original approach, guided self-detox can be achieved more easily if other lifestyle changes are taking place at the same time, and family and important others (if present) are aware and supportive of the plan. These are the other two crucial components of SPADe. Early and gradual implementation of changes within the individual's lifestyle are necessary to provide: (i) a routine in everyday life that would protect from early relapse; (ii) fill in the void that alcohol detoxification would leave behind; (iii) could be used as distraction strategies against cravings; (iv) would enhance personal responsibility; (v) would de-mystify alcohol and challenge the omnipotence of cravings or withdrawal symptoms, and finally; (vi) would protect from the acute stress experienced in the early days of abstinence. According to SPADe, these lifestyle changes should be initiated and tested while alcohol is stabilized and to be augmented, as well as evaluated, after the detoxification. The involvement of family members and the immediate social support system helps by providing education, modifying unrealistic expectations, and supporting a more gradual adaptation to the new family dynamics (following the removal of alcohol). It helps with managing anxiety and the difficult feelings/emotions associated with broken trust and promotes a partnership approach. The fundamental reason for this involvement is that recovery is easier and more sustainable within a respectful, stress-free, and supportive environment. These lifestyle changes and possible family involvement should be discussed in depth with the individual as they might be particularly challenging due to social restrictions associated with COVID-19.

### Managed Alcohol Programs (MAPs)

MAPs go beyond tolerance of alcohol onsite in housing or other accommodation. MAPs are a strategy to assist people to manage their alcohol use with the aim of reducing harms of consumption, including consumption of non-beverage alcohol (80). In Canada, we witnessed the growth and implementation of many new MAPs with the onset of COVID 19. The need for MAPs during COVID-19 was a strategy to assist with physical distancing and self-isolation by reducing the need for participants to source alcohol daily, as well as reducing risks of withdrawal and avoiding use of non-beverage alcohol and substitution of illicit drugs associated with high rates of overdose deaths. In British Columbia, specific operational guidance was released to assist with the development of a range of models (81).

MAPs originated as a response to the complex needs of people who do not respond to abstinence programs and are experiencing homelessness or housing instability (82). A maximum number of doses are provided to participants daily. MAPs intend to replace non-beverage alcohol, heavy drinking episodes, and intoxication, with a steady source of alcohol, and thereby reduce acute alcohol-related harms (82). To the extent that MAPs contribute to reductions in total alcohol consumption among people with AUDs who are not willing or able to abstain, they may also contribute to lower risks of serious alcohol-related diseases, though these will still be high compared to general population (83). MAPs offer regulated access to beverage alcohol, alongside meals, healthcare, accommodation and a range of social supports. There are a wide range of models, from community programs led by people with lived experience, to programs in shelters, transitional and supportive housing, and hospitals. Despite the range in models, the goals of MAPS are to reduce harms and provide an option for those who have not been successfully supported by other approaches and do not wish to stop drinking. MAPS seek to provide an alternative to street-based survival drinking and/or use of non-beverage alcohol. An important element of MAPs, consistent with a harm reduction framework, is the involvement of people with lived experience in design, development, and delivery of programs (53, 54, 84, 85).

Podymow and colleagues first documented the impacts of MAPs in 2006, based on a program in Ottawa, and found benefits related to reduced hospital and policing costs, improved hygiene and nutrition, and increased medication compliance (82). The Canadian Managed Alcohol Study (CMAPS) began in 2011 and is the largest study to date of MAP implementation and outcomes (www.cmaps.ca). Initial studies of MAPs found evidence of reduced alcohol-related harms, reduced use of non-beverage alcohol, improved quality of life and safety, increased housing stability, and reduced demands and costs for the health and criminal justice systems (86–88). Management issues related to eligibility criteria, and tailoring programs to individual needs, were identified. In a comparison of 175 MAP participants and 189 controls in five cities, Stockwell et al. found that long-term MAP residents (>2 months) drank significantly fewer drinks per day than controls over the previous 30 days (83). In this same analysis, long-term MAP residents reported significantly fewer acute alcohol-related harms in the domains of health, safety, social, legal, and withdrawal symptoms. The same participants reported that, when unable to afford alcohol, they would often use positive coping strategies e.g., waiting for money (46%), make supplies last longer (53%), seek treatment (37%) or go without alcohol (39%), and be less likely to use strategies with negative or harmful consequences, such as use illicit drugs (usually cannabis) (28%) and/or non-beverage alcohol (30%) (75). Compared to controls, the long-term MAP residents were significantly less likely to use illicit substances, steal, or go without alcohol, and they were more likely to seek treatment. In the first longitudinal analysis of 59 MAP participants and 116 controls, Stockwell et al. (89), found that MAP participants drank less hazardously than controls and experienced fewer alcohol related harms at 0–6 months than controls (89). Additionally, qualitative findings from MAP participants suggested that being in a MAP disrupts survival drinking and cycling through multiple settings (which is particularly important to reduce movement in the context of COVID-19), as well as enhancing feelings of safety, belonging, sense of place or home, and hope for the future (90). This evidence indicates that acute and social harms (e.g., injuries, poisoning) can be reduced for this population by engagement in a MAP. In order to reduce chronic harms, and elevated risk of alcohol-related diseases created by a program of continuous daily alcohol administration, attention to program policies and administration is critical (83, 89).

In summary, MAPs have been shown to enhance housing stability, reduce acute and social alcohol-related harms, improve safety, and create opportunities for reconnection with families, communities, and healing. However, there has been limited research on programs that incorporate sex and gender considerations, or the needs of ethnically diverse populations as the majority of the existing programs primarily serve men. A recent study conducted in Scotland that aimed to explore the potential of MAPS concluded that the model held much promise for implementation across Scotland and potentially in the UK more widely, and recommended that they should be taken forward into pilot implementation (63). MAPs fill an important gap for those who require additional support to manage alcohol use in order to maintain stability and, during COVID-19, adhere to stay at home and physical distancing measures.

## Discussion

Services for people with AUD have largely focused on treatment approaches that have a goal of abstinence. Arguments for the appropriateness of harm reduction strategies for the most vulnerable subgroup of people with AUD, namely people who are homeless, is not new (7, 91). Implementation of public health measures to reduce the spread of COVID-19 have increased alcohol consumption in some countries (32), especially those that have relaxed policies on alcohol availability and pricing. This has added to pressures on service provision for people with AUD and highlighted the need for new approaches during a pandemic (92, 93). Reductions in, and substantial limitations of, provision of services to this group has created an opportunity for a further shift in philosophy toward harm reduction in substance use services, as well as implementation of services that focus on substitution, tolerance, and safer or managed use of alcohol. Medications that help to manage alcohol craving or withdrawal are often used when the goals are for abstinence, while cannabis substitution may provide a less harmful substance to replace alcohol. In addition, safer drinking education (e.g., about lower risk beverages, contexts and drinking patterns) is a harm reduction strategy that has been incorporated into Housing First initiatives but could be provided in other community settings. In this paper, we have discussed the strategic need and evidence for the enhancement of treatment services through the explicit incorporation of alcohol harm reduction approaches both during the COVID-19 period and beyond.

While there is a growing evidence base for alcohol harm reduction beyond population level policies that seek to reduce overall harms, we recognize that the incorporation of alcohol harm reduction approaches described here require philosophical shifts as well as policy shifts. Our view is that such shifts, and the associated change of attitudes toward one of the most vulnerable and marginalized groups in society, would contribute toward the reduction of discrimination and systemic neglect of their needs. To be clear, we are not proposing that all services be oriented to harm reduction but, rather, that harm reduction be a recognized and accepted approach within mainstream substance use services in order to expand access to a broader range of services based on client choice and goals. It is the underlying values base of harm reduction in which the explicit intention is to reduce harm that has created controversy as it conflicts with long established and often dominant norms of abstinence as the ultimate goal of substance use services for people with AUD. Paradoxically there are individuals often impacted by structural inequities and vulnerability who are left out or even potentially impacted by unintended consequences of such policies (13).

In this position paper, we have specifically examined approaches that provide alcohol within a harm reduction framework, namely SPADe and MAPs. While both provide alcohol, and share goals related to reducing harm through provision of a safe and regular source of alcohol to address harms of binging and smoothing out of drinking patterns, there are differences between the two approaches. The ultimate aim of SPADe is abstinence, while MAPs aim to reduce harms as a primary goal with or without necessarily resulting in eventual abstinence. Both take a pragmatic and incremental approach to provision of alcohol which is aligned with harm reduction principles more generally (8). Our view is that there is much that can be learned from both approaches in meeting the needs of clients with severe AUD. For example, MAPs might incorporate elements of SPADe for clients who express an interest in reducing alcohol consumption and/or goals of abstinence. Alternatively, SPADe programs may identify clients who would be better suited to MAPs. As such, the existence of such programs provides an expanded range of services for those with severe AUD who are often overlooked or underserved by current treatment systems.

## Conclusion

Alcohol harm reduction that spans tolerance of ongoing drinking and provision of alcohol, as well as substitution, have become more important during COVID-19. However, such approaches have a history preceding COVID-19 and a place in the broader landscape of harm reduction that is often dominated by illicit drugs. While COVID-19 has dramatically reduced and limited services, the pandemic has propelled the importance of alcohol harm reduction and created new opportunities for implementation of harm reduction philosophy and approaches, including programs that incorporate the provision of alcohol as medicine as part of the substance use treatment continuum.

## Author Contributions

All authors listed have made a substantial, direct and intellectual contribution to the work, and approved it for publication.

## Conflict of Interest

The authors declare that the research was conducted in the absence of any commercial or financial relationships that could be construed as a potential conflict of interest.
